# Sorafenib improves lipiodol deposition in transarterial chemoembolization of Chinese patients with hepatocellular carcinoma: a long-term, retrospective study

**DOI:** 10.18632/oncotarget.18811

**Published:** 2017-06-29

**Authors:** Lin Zheng, Chen-Yang Guo, Cheng-Shi Chen, Jin-Cheng Xiao, Hong-Tao Hu, Hong-Tao Cheng, Deng-Wei Zong, Li Jiang, Hai-Liang Li

**Affiliations:** ^1^ Department of Radiology, Zhengzhou University Affiliated Cancer Hospital, Zhengzhou 450008, China

**Keywords:** lipiodol deposition, hepatocellular carcinoma, TACE, sorafenib, overall survival

## Abstract

**Objective:**

Though synergy of sorafenib and transarterial chemoembolization (TACE) in hepatocellular carcinoma (HCC) is well discussed in previous reports, association of lipiodol retention by sorafenib addition to TACE with the survival outcomes remain elusive. Therefore, we studied the impact of sorafenib addition to TACE on survival outcomes mediated by lipiodol retention.

**Materials and Methods:**

This is a long-term, retrospective, single-center study using medical records of patients diagnosed with HCC at the Department of Interventional Radiology of Zhengzhou University Affiliated Cancer Hospital (China) between April 2004 and March 2012.

**Results:**

Lipiodol deposition of > 50% was significantly increased in TACE + sorafenib group (70.87%) compared to TACE alone group (45.11%) (P = 0.0001). Significant increase in lipiodol deposition with sorafenib treatment was observed compared to TACE alone group (OR = 0.449, P = 0.041). The median overall survival in TACE + sorafenib and TACE alone groups were 38 months [95% CI = 9.772–56.228] and 31 months [95% CI = 21.855–40.145] respectively. Also, the hazard of death was comparatively greater in TACE alone group than TACE + sorafenib group [HR = 1.071]. Response rate to the therapy significantly increased after sorafenib administration to TACE patients, [compared to TACE alone treatment [69/103 (66.99%)] vs 55/133 (41.35%)], P = 0.0001.

**Conclusions:**

Lipiodol deposition is significantly increased upon sorafenib addition after TACE. However, there was no significant impact of lipiodol deposition on the survival benefits exerted by the synergistic combination and hence, future prospective trails are warranted to validate the findings of this study.

## INTRODUCTION

Hepatocellular carcinoma [HCC] is the seventh most frequent cancer in males and ninth most common in females, leading to more than 500 000 deaths annually throughout the world [1]. China alone accounts for 53.5% of HCC cases worldwide [2]. Though tumor resection and liver transplant are the current therapeutic approaches employed to treat HCC, these may be restricted to patients with ill preserved liver functions such as, presence of liver cirrhosis, hypertension portal, coagulopathy, increased bilirubin or hepatic dysfunction [3].

Eventually, transarterial chemoembolization [TACE] has gained wide acceptance as standard of care for treating unresectable HCC [4]. Conventional TACE therapy involves intra-arterial delivery of chemotherapeutic agents along with lipiodol followed by injection of embolizing agents like gelatin sponge particles to necrotize the tumor tissue [5]. Though lipiodol is used as a chemo drug eluting substance in TACE, it itself has tumor necrotizing ability. Exogenous Lipiodol administered after resection has also shown increased recurrence free survival in HCC patients [6]. Recent reports on meta-analysis of controlled trials on TACE therapy for HCC claimed that, addition of a chemotherapeutic agent did not exert extra benefit to increase the overall survival (OS) compared to the transarterial embolization [TAE] [7]. Previous studies reported that, poor lipiodol retention by the tumor is a major factor affecting OS and time to progression (TTP) associated with TACE therapy. Further, the risk of disease progression in poor lipiodol retention is negatively correlated with increased TACE interventions and degree of lipiodol deposition after the first TACE [8].

The addition of an anti-angiogenic, vascular endothelial growth factor (VEGF) inhibitor sorafenib, to TACE has been experimented in the recent past to increase the OS and TTP in HCC patients. Several studies have reported the efficiency of sorafenib to prolong OS of HCC patients in combination with TACE [9–13]. Moreover, sorafenib was well tolerated and improved OS after resection in Barcelona Clinic Liver Cancer (BCLC) stage C hepatocellular carcinoma [14] and in patients’ refractory to TACE [15]. It has also proven efficiency in preventing HCC recurrences post liver transplantation [16]. Most of all, sorafenib also increases average interval and frequency of TACE, thereby paving high chances for lipiodol retention by the tissue thereby achieving anti-tumor effect [17].

Consequently, it can be hypothesized that the beneficial effects of sorafenib along with TACE therapy in HCC may be related to the lipiodol deposition. Till date there are no studies documented on the effect of sorafenib on lipiodol deposition. In this study, we have evaluated the lipiodol retention ability of sorafenib and compared the OS and TTP of HCC between TACE alone and TACE + sorafenib treatment groups.

## RESULTS

### Demographic characteristics of the patients

A total of 236 patients were included in the study. Of which, TACE group had 133 patients (males, *n =* 117; females, *n =* 16) and TACE + sorafenib group had 103 patients (males, *n =* 88; females, *n =* 15). Mean age of patients in TACE group was 57.35 ± 11.88 years and TACE + sorafenib group was 53.88 ± 12.25 years. There were no statistical differences in the serum level of AFP and HbsAg, Eastern Cooperative Oncology Group (ECOG) performance status, BCLC staging, number of tumors and frequency of TACE between the two groups, except for the Child-Pugh class categories A, B and C, where significant difference between the TACE and TACE + sorafenib group was observed (*P* < 0.0001) (Table 1).

**Table 1:** Baseline characteristics of HCC patients receiving TACE and TACE + sorafenib

Baseline characteristics	TACE + sorafenib	TACE	*P*-*value*
Gender			
Male, *n* (%)	88 (85.44)	117 (87.97)	0.5678
Female, *n* (%)	15 (14.56)	16 (12.03)	
Age			
Mean ± SD [median]	53.88 ± 12.25	57.35 ± 11.88	0.0291
Child-Pugh class			
A, *n* (%)	45 (43.69)	127 (96.21)	< 0.0001
B, *n* (%)	53 (51.46)	5 (3.79)	
C, *n* (%)	5 (4.85)	0 (0.00)	
AFP			
< 200, *n* (%)	55 (53.40)	59 (45.04)	0.2041
> = 200, *n* (%)	48 (46.60)	72 (54.96)	
ECOG			
0, *n* (%)	52 (50.48)	75 (56.39)	0.484
1, *n* (%)	33 (32.03)	58 (43.61)	
2, *n* (%)	18 (17.47)	0 (0.00)	
BCLC stage			
B, *n* (%)	68 (66.02)	85 (63.91)	0.7364
C, *n* (%)	35 (33.98)	48 (36.09)	
HbsAg			
Negative, *n* (%)	6 (5.83)	10 (7.52)	0.6078
Positive, *n* (%)	97 (94.17)	123 (92.48)	
Iodine oil deposit			
< 50%, *n* (%)	30 (29.13)	73 (54.89)	0.0001
> = 50%, *n* (%)	73 (70.87)	60 (45.11)	
Tumor size			
< 7 cm, *n* (%)	65(65.66)	133(100.0)	0.0000
> = 7 cm, *n* (%)	34(34.34)	0 (0.00)	
No. of tumors			
< 3, *n* (%)	58 (58.59)	77 (58.33)	0.9693
> = 3, *n* (%)	41 (41.41)	55 (41.67)	
TACE frequency			
< 3, *n* (%)	67 (65.69)	92 (69.17)	0.5712
> = 3, *n* (%)	35 (34.31)	41 (30.83)	

### Comparison of Lipiodol deposition profile of TACE and TACE + sorafenib groups

**Figure 1:**
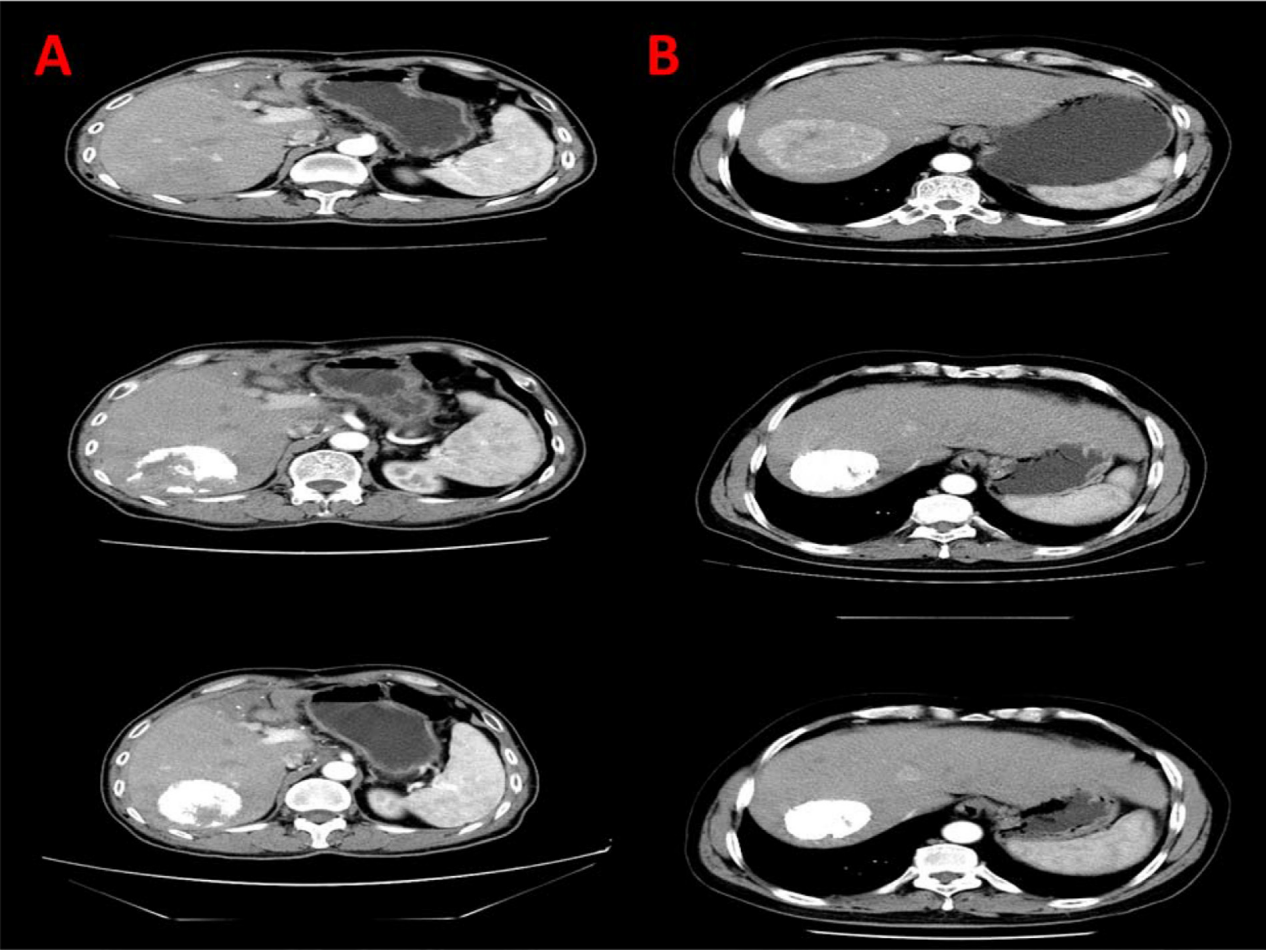
CT scans depiction of lipiodol deposition in TACE alone and TACE + sorafenib group (**A**) Lipiodol deposition in the right lobe of liver in TACE alone group. (**B**) Increased lipiodol deposition in the right lobe of liver in TACE + sorafenib group.

Iodine oil/ Lipiodol deposition was a short-term outcome measured by analyzing the computer tomography (CT) scan images of patients (Figure 1). Iodine oil deposition of > 50% [8] was significantly increased in TACE + sorafenib group (70.87%) compared to TACE alone group (45.11%) (*P* = 0.0001) (Table 1). Upon logistic regression analysis of the two groups we found that sorafenib treatment in patients receiving TACE significantly increased lipiodol deposition (OR = 0.449, *P =* 0.041) (Table 2).

**Table 2:** Factors affecting lipiodol deposition

Independent variable	*P*-value	OR	95% CI	
Age	0.627	1.006	0.983	1.029
Gender	0.756	1.147	0.483	2.722
ECOG	0.582	0.857	0.495	1.484
Child-Pugh class	0.252	1.585	0.721	3.486
BCLC	0.248	0.675	0.347	1.314
HbsAg	0.815	0.869	0.268	2.817
AFP	0.111	0.631	0.358	1.112
Tumor size	0.679	1.236	0.453	3.367
No. of tumors	0.486	0.812	0.453	1.457
TACE times	0.958	0.984	0.532	1.820
GROUP	0.041	0.449	0.208	0.968
Constant	0.309	4.706		

Dependent variable: lipiodol Deposit (0 =< 50%, 1 =>= 50%).

Independent variable: Gender: 1 = Male, 2 = Female; Child-Pugh class: 1 = A, 2 = B, 3 = C; BCLC: 1 = B, 2 = C; sAg: 1 = Positive, 0 = Negative; Group: 1 = TACE+sorafenib, 2 = TACE;

AFP: 0 =< 200, 1 =>= 200; Tumor size: 0 =< 7 cm, 1 =>= 7 cm; No. of tumors: 0 =< 3, 1 =>=3;

TACE times: 0 =< 3, 1 =>= 3.

### Logistic regression analysis of factors associated with Lipiodol deposition

Logistic regression showed lipiodol deposition was not significantly affected by increase in age and gender (OR = 1.006, *P* = 0.627 and OR = 1.147, *P* = 0.756 respectively). Whereas, lipiodol deposition was slightly increased with increased number of TACE interventions (< 3 TACE vs. ≥ 3 TACE; OR = 0.984), less number of tumors (< 3 vs. ≥ 3; OR = 0.812, *P* = 0.958) and slightly decreased with increased tumor size (< 7 cm vs ≥ 7 cm; OR = 1.236, *P* = 0.679). Lipiodol deposit was further decreased with the presence of Child-Pugh C class (OR = 1.585, *P* = 0.252) and increased with BCLC stage B over C (OR = 0.675, *P* = 0.248), ECOG status (OR = 0.857, *P* = 0.582) and HbsAg status (OR = 0.869, *P* = 0.815). None of these impacts were statistically significant (Table 2).

### Tumor response evaluation according to modified response evaluation criteria in solid tumors (mRECIST)

Tumor responses were quantified by observing the CT scans of patients of two groups. Responders (complete response (CR) + partial response (PR)) to the treatment were significantly increased after sorafenib administration to TACE patients, [69/103 (66.99%)] compared to TACE alone treatment [55/133 (41.35%)], *P* = 0.0001. None of the patients reached CR in the TACE alone group and majority of the patients developed stable disease. Details of Tumor response evaluated as per mRECIST are shown in Table 3.

**Table 3:** Tumor response evaluation by mRECIST

MRECIST criteria	TACE + sorafenib	TACE	χ^2^	*P*
CR, *n* (%)	2 (1.94)	0 (0.00)		
PR, *n* (%)	67 (65.05)	55 (41.35)	15.300	0.0001
SD, *n* (%)	28 (27.18)	68 (51.13)		
PD, *n* (%)	6 (5.83)	10 (7.52)		

### Comparison of time to event rates between TACE alone and TACE + sorafenib groups

**Figure 2:**
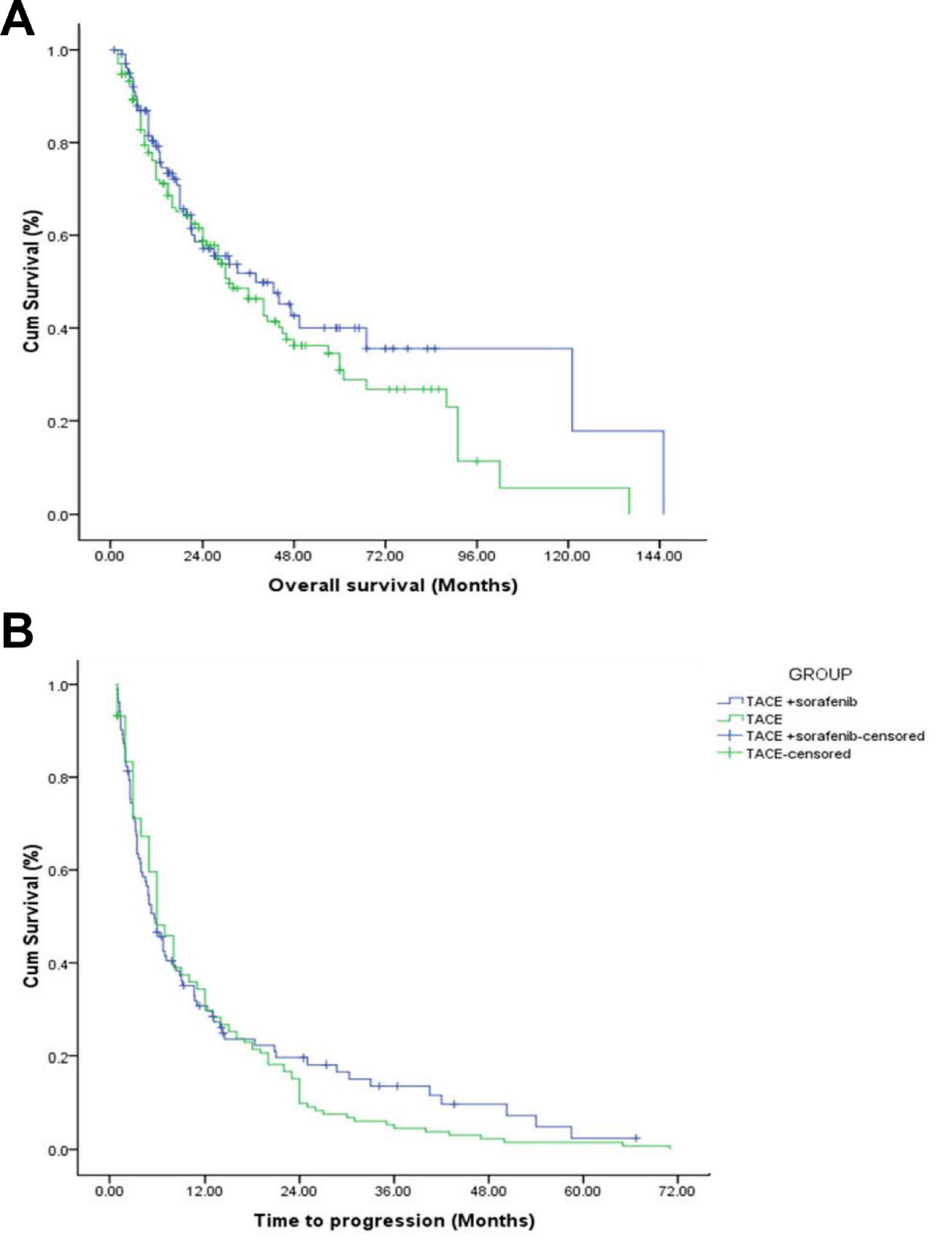
OS and TTP curves of TACE and TACE + sorafenib group

Kaplan Meir curves were constructed for OS and TTP determination and are depicted in Figure 2A–2B respectively. 129/236 patients had died at the time of OS data analysis. The median OS of all the remaining 107 patients was 33.1 months [95% CI = 24.263–41.937]. The median OS in TACE + sorafenib and TACE alone groups were 38 months [95% CI = 9.772–56.228] and 31 months [95% CI = 21.855–40.145] respectively. The log rank test did not reveal significant difference between the two groups, *P* = 0.254. The hazard of death was comparatively greater in TACE alone group than TACE + sorafenib group [HR = 1.071; *P* = 0.816, non-significant] as observed in the cox proportional hazard analysis (Table 4). Similarly, the median TTP duration in TACE + sorafenib and TACE alone groups were 5.7 months [95% CI = 4.243–7.157] and 6 months [95% CI = 5.2–6.7] respectively. However, the log rank test did not reveal significant difference between the two groups, *P* = 0.645. The hazard of disease progression was comparatively greater in TACE alone group than TACE + sorafenib group [HR =1.329; *P* = 0.164, non-significant] as observed in the cox proportional hazard analysis (Table 4).

**Table 4:** Cox proportional hazards model OS and TTP

Independent variable	OS				TTP			
	*P*-value	HR	95% CI		*P*-value	HR	95% CI	
Age	0.354	1.008	0.991	1.024	0.630	1.003	0.992	1.014
Gender	0.622	1.164	0.637	2.127	0.226	1.317	0.844	2.055
ECOG	0.194	0.777	0.531	1.137	0.470	1.112	0.834	1.484
Child-Pugh class	0.108	0.633	0.363	1.105	0.873	1.035	0.677	1.583
BCLC	0.002	1.956	1.292	2.959	0.634	0.924	0.667	1.280
HbsAg	0.188	0.632	0.320	1.251	0.530	0.832	0.468	1.478
GROUP	0.816	1.071	0.601	1.910	0.164	1.329	0.890	1.984
Iodine oil deposit	0.923	0.981	0.665	1.447	0.392	0.880	0.656	1.180
AFP	0.037	1.491	1.024	2.171	0.438	1.119	0.843	1.486
Tumor size	0.005	2.485	1.310	4.715	0.017	1.834	1.115	3.016
No. of tumors	0.033	1.517	1.033	2.227	0.138	1.254	0.930	1.692
TACE times	0.014	0.591	0.388	0.900	0.193	0.817	0.602	1.108

Gender: 1 = Male, 2 = Female; Child-Pugh class: 1 = A, 2 = B, 3 = C; BCLC:1 = B, 2 = C; HbsAg:1 = Positive, 0 = Negative; Group: 1 = TACE + Sorafenib, 2 = TACE; Iodine Oil Deposit: 0 =< 50%, 1 =>= 50%; AFP: 0 =< 200, 1 =>= 200; Tumor size: 0 =< 7cm, 1 =>= 7 cm; No. of tumors: 0 =< 3, 1 =>= 3; TACE times: 0 =< 3, 1 =>= 3.

### Cox proportional analysis to determine factors associated with OS and TTP

Cox proportional analysis showed that the hazard of death increased significantly in subjects with BCLC stage C compared to stage B [HR = 1.956; *P* = 0.002]; increase in AFP [HR = 1.491; *P* = 0.037]; and increased number of tumors [HR = 1.517, *P* = 0.033]. The hazard significantly decreased in patients with increased TACE interventions [HR = 0.591, *P* = 0.014] and non-significantly with increase in lipiodol oil deposition [HR = 0.981, *P* = 0.923]. The hazard of disease progression/TTP significantly increased with increased tumor size [HR = 1.834, *P* = 0.017]. Other factors such as age, gender, ECOG status, Child-Pugh class, HbsAg level had non-significant impact on OS and TTP (Table 4).

### Safety events

Among the major adverse reactions associated with TACE plus sorafenib treatment and TACE alone treatment, occurrence of biloma with abscess was more in TACE treatment group (3 patients, 2.3%) when compared to TACE-sorafenib group (1, 1%; Table 5). Significant increase in minor adverse events (abdominal pain, fever, vomiting) were observed in TACE alone therapy than TACE-sorafenib combination therapy. In the TACE- sorafenib combination treatment, the rate of occurrence of low grade (grade 1–2) sorafenib-related adverse events (hand-foot skin reaction, diarrhea, fatigue, hypertension, rash, alopecia and voice changes) was more. Grade 3 reactions were observed in hand-foot skin reactions (9(8.7%)) and hypertension (2(1.9%)) and no grade 4 reactions were reported (Table 6).

**Table 5:** Adverse events related to TACE for the two treatment groups

Adverse events	TACE + sorafenib *n =* 103, *n* (%) *n* (%)	TACE *n =* 133	*X*^*2*^ value	*P*-value
Major Adverse Events				
Upper GI bleeding	2 (1.9)	2 (1.5)		1.000^*^
Biloma with abscess	1 (1.0)	3 (2.3)		0.634^*^
Minor Adverse Events				
Abdominal pain	74 (71.8)	91 (68.4)	0.323	0.570
fever	73 (70.9)	98 (73.6)	0.688	0.407
vomiting	41 (39.8)	59 (44.4)	0.493	0.482
1-2 myelosuppression	4 (3.8)	5 (3.7)		1.000^*^

TACE, transcatheter arterial chemoembolization; GI, gastrointestinal; ^*^Fisher’s Exact Test was used.

**Table 6:** Incidence of sorafenib-related adverse events in the TACE-sorafenib group

Adverse events	All grade,	Grade 1-2,	Grade 3,	Grade 4,
	*n* (%)	*n* (%)	*n* (%)	*n* (%)
Hand-foot skin reaction	83 (80.6%)	74 (71.8%)	9 (8.7%)	0
Diarrhea	66 (64.1%)	66 (64.1%)	0	0
Fatigue	25 (24.3%)	25 (24.3%)	0	0
Hypertension	9 (8.7%)	7 (6.8%)	2 (1.9%)	0
Rash	8 (7.8%)	8 (7.8%)	0	0
Alopecia	22 (21.4%)	22 (21.4%)	0	0
Voice changes	2 (1.9%)	2 (1.9%)	0	0

## DISCUSSION

In our last retrospective study, TACE effect was proved to be significantly associated with lipiodol retention [8]. Many other studies have also reported that TACE deposition correlates well with tumor necrosis [18–20]. In addition, sorafenib addition to TACE therapy is recommended as an effective therapy for advanced HCC [9–16]. Nevertheless, the underlying mechanism of the synergistic effect were not understood.

HCC is one of the highly-vascularized tumors. Lipiodol is selectively uptaken and retained in hyperarterialyzed liver tumors thereby improving the pharmacokinetics of the drug and tumor response [21]. Circulating VEGF is associated with tumor progression in most of the tumors [22, 23]. Xin et al. reported that pre-TACE circulating VEGF levels were significantly high in HCC patients and the levels were considerably decreased post TACE treatment. This further confirms the role of circulating VEGF in HCC also [24]. Moreover, circulating VEGF levels were associated with poor outcomes in patients with diversified tumors [25–28]. Unambiguously, increase in VEGF correlated with decreased lipiodol retention in HCC patients suggesting that VEGF ate up the lipiodol deposited in the tumor [24]. Additionally, in a study conducted to assess the correlation between increased VEGF levels and prognosis after TACE in HCC patients, lipiodol deposition was significantly increased in untreated HCC patients whereas lipiodol deposition increased in 1–2 days after they received first TACE [29]. The reason why we chose iodine oil/lipiodol deposit as the short-term endpoint is due to the assumption that hyper vascular progressing tumor usually washes out deposited lipiodol. As sorafenib can delay tumor progression, the addition of sorafenib to TACE could reduce the consumption of lipiodol. Sorafenib may reduce endothelial nitric oxide synthase (eNOS) activity by inhibiting vascular endothelial growth factor receptors (VEGF-R), leading to a decrease in nitric oxide production and increase lipiodol retention. In the present study, lipiodol has not shown correlation with the survival outcomes associated with the combination treatment, but we find some evidence that increased lipiodol deposition may increase the anti-VEGF activity of sorafenib as lipiodol retention increases the tumor vascular permeability of the drug and hence, increased anti-VEGF activity may be the reason behind the survival benefits. Till date the correlation between lipiodol deposition and sorafenib effect in combination with TACE in HCC is obscure. There is evidence for the antiangiogenic effect mediated efficacy of sorafenib but here we aimed to see if the antiangiogenic property of sorafenib is boosted by increasing the lipiodol retention. As this is a preliminary study on lipiodol retention, we did not show the correlation of lipiodol retention with increased anti-VEGF property and hence a further study on the mechanistic role of lipiodol retention may favor the findings of the present study. The present study findings itself may prove as pioneer evidence for further studies.

Overall our retrospective study revealed a significant increase in lipiodol deposition with addition of sorafenib to TACE compared to TACE alone, proving a synergistic effect of the combination. This may be attributed to the anti-VEGF effect of sorafenib as discussed above. Upon logistic regression, we found that lipiodol deposition was slightly increased with increased number of TACE interventions, decreased number of tumors and BCLC stage B over C, whereas, slightly decreased with increased tumor size, presence of Child-Pugh C class, ECOG and HbsAg status which are in line with our previous reports [8]. None of these impacts were statistically significant, and this may be due to high heterogeneity among the patients and also small sample size and retrospective study nature. Though several meta-analyses of major RCTs showed a superior clinical efficacy of sorafenib addition to TACE therapy over TACE alone therapy, the OS differed tremendously among different patient population, irrespective of the treatment pattern. In a meta-analysis of five comparative studies (2 RCTs) by Wang et al. [30] with a total of 899 patients, though sorafenib increased OS in TACE treated patients the difference was not significant when compared with TACE alone. However, in an earlier meta-analysis of six published studies with a total of 1254 patients a significant difference in OS improvement was seen between the two groups [31]. Nevertheless, a larger meta-analysis on nine studies but with a lower sample size (*n =* 900), did not yield significant PFS between the groups but however showed a downward trend in progression free rate [32]. From this one may infer that, the response to the treatments vary among patients tremendously and depends on the study design and sample size. Larger sample size and RCT design yielded more significant results than other comparative non-RCT studies with smaller sample size. Nevertheless, the non-significant findings had an impact on the clinical efficacy of the treatment. Our study also showed improved OS and TTP in HCC patents, though significant impact of lipiodol deposition on the OS and TTP was not observed. There may be other reasons which may decide the OS and TTP apart from lipiodol deposition in TACE + sorafenib and hence, we restrict our study to the association of lipiodol retention with sorafenib and its synergy with TACE. Also, researchers suggest that TACE plus sorafenib therapy was well tolerated and gave better survival outcomes in advanced HCC patients and hence, as this study involved intermediate stage HCC patients future studies are warranted to delineate the involvement of lipiodol retention on OS and TTP in advanced HCC patients and that the results from this study may be preliminary and an insight that lipiodol retention is improved with sorafenib and further randomized trials are warranted to evaluate the impact of the lipiodol deposition on survival benefits. One more reason for non-significant association of lipiodol deposition with survival may be due to the ethnicity of patients. In previous studies, sorafenib did not significantly prolong TTP or OS in Asian patients compared to non-Asians with HCC who responded to TACE [33]. Moreover, the incidences of adverse events were also worsened in the patients treated with sorafenib and TACE combination therapy [34]. The complication profile of the therapy reported in previous studies was similar with that of our study. Further, studies by Iavarone et al. [35] and Abou-Alfa [36], reported treatment interruptions and dose changes along with high adverse events in patients who responded to TACE and were treated with sorafenib and that factors such as ECOG status, macrovascular invasion, extrahepatic spread of tumor were independent predictors of survival. Similar way, the lipiodol retention may be influenced by similar predictors and might have led to non-significant association with OS.

To conclude, lipiodol deposition is significantly increased upon sorafenib addition after TACE. However, there was no significant impact of lipiodol deposition on the survival benefits exerted by the synergistic combination and hence, future prospective trails are warranted to validate the findings of this study. Also, the synergy of the combination may be correlated with the anti-VEGF effect of sorafenib which needs further validation.

## MATERIALS AND METHODS

### Study design and patient eligibility

We performed this long-term, retrospective, single-center study by evaluating medical records of subjects who were diagnosed with HCC at the Department of Interventional Radiology of Zhengzhou University Affiliated Cancer Hospital, China between April 2004 and March 2012. Patients diagnosed with HCC (BCLC stage B or C), Child-Pugh grade A, B or C, ECOG score of 0, 1 and 2, those received at least two cycles of TACE assigned to sorafenib (400 mg, twice daily (BID)) group or control group and patients with reported data on iodine oil/lipiodol deposition and efficacy and/or safety analyses were included in the study. Patients previously treated with microwave ablation, radiofrequency ablation, surgical resection or liver transplantation after TACE, platelet count < 50 × 10^9^/L were excluded from the study. Participants with missing data were also excluded automatically. Informed consent was not a prerequisite since this was a retrospective study. The patients’ information was anonymized throughout the study for confidentiality.

HCC patients were assigned to two cohorts based on the interventions they received, into 1) TACE alone group and; 2) TACE + sorafenib group; in this group sorafenib 400 mg BID was initiated within 1 week before the first TACE. The CT examinations were performed within 1 week of each TACE cycle. The interval between each treatment cycle was 1 month. As lipiodol is retained in the tumor nodules up to 1-year after injection and has also reported immediate retention with a CT examination performed within 7–14 days after TACE [37–39], we performed the CT early for detecting the early retention of lipiodol.

### Study outcomes and safety assessment

Demographic characteristics of patients were obtained from medical records. Primary outcome of the study was to quantify and compare lipiodol deposition in TACE alone and TACE + sorafenib cohorts. Lipiodol deposition was evaluated using CT scans and it was quantified as a measure of increased tumor area density. The denser the tumor area, the maximum is the lipiodol deposition. Secondary outcomes were assessment of OS (defined as the time interval from when the intervention was approved for the patient to death from any cause) and TTP (defined as time from randomization until tumor progression, not including death).

We also examined tumor responses using modified response evaluation criteria in solid tumors (mRECIST) which include CR, disappearance of all clinical evidence of disease), PR (at least 30% reduction in size of all measurable tumors), stable disease (SD, Between a 30% reduction or < 25% increase in the size of all detectable tumors), and progressive disease (PD, Patients or proportion of patients with a ≥ 25% increase in size of tumors since previous measurement) rates, as the secondary outcome for the patients treated with this combination therapy. Response rate was defined as the proportion of patients with CR and PR in the analyzed population.

### Statistical analysis

Patient demographics, clinical history, laboratory data, and CT scan images were collected. Logistic regression analysis was performed on independent variables that would impact lipiodol deposition included age, gender, ECOG, Child-Pugh class, BCLC stage, HbsAg and AFP levels, tumor size, number of tumors and frequency of TACE. Descriptive analysis was performed for demographic characteristics and the populations were represented as counts and percentages. Kaplan-Meier curves were drawn to determine the survival rates (OS) and TTP in subjects of both experimental groups. Cox Proportional Hazard Regression was used for a multivariable adjusted analysis of survival curves with demographic and clinical baseline characteristics. HCC tumor necrosis was measured from CT scan data according to mRECIST criteria. We utilized Chi-square analysis to test association between mRECIST and categorical variables. All statistical analyses were performed with the IBM SPSS statistical software version 22 for Windows (IBM Corp., Armonk, New York, USA). A two-tailed *p* of < 0.05 was considered significant.
